# Long-term Follow-up After Aneurysm Treatment with the Flow Redirection Endoluminal Device (FRED) Flow Diverter

**DOI:** 10.1007/s00062-023-01346-3

**Published:** 2023-10-13

**Authors:** Sophia Hohenstatt, Christian Ulfert, Christian Herweh, Tim Hilgenfeld, Niclas Schmitt, Silvia Schönenberger, Min Chen, Martin Bendszus, Markus A. Möhlenbruch, Dominik F. Vollherbst

**Affiliations:** 1 Department of Neuroradiology, Heidelberg University Hospital, INF 400, 69120 Heidelberg, Germany; 2 Department of Neurology, Heidelberg University Hospital, Heidelberg, Germany

**Keywords:** Cerebral aneurysm, Flow diverter stent, Long-term outcome, Embolization, Intraluminal device

## Abstract

**Introduction:**

This study focuses on long-term outcomes after aneurysm treatment with either the Flow Re-Direction Endoluminal Device (FRED) or the FRED Jr. to investigate the durability of treatment effect and long-term complications.

**Methods:**

This study is based on a retrospective analysis of a prospectively maintained patient data base. Patients treated with either FRED or FRED Jr. between 2013 and 2017 at our institution, and thus a possibility for ≥ 5 years of follow-up, were included. Aneurysm occlusion rates, recurrence rates, modified Rankin scale score shifts to baseline, and delayed complications were assessed.

**Results:**

In this study 68 patients with 84 aneurysms had long-term follow-up with a mean duration of 57.3 months and 44 patients harboring 52 aneurysms had a follow-up ≥ 5 years with a mean follow-up period of 69.2 months. Complete occlusion was reached in 77.4% at 2 years and increased to 84.9% when the latest available imaging result was considered. Younger age and the absence of branch involvement were predictors for aneurysm occlusion in linear regression analysis. After the 2‑year threshold, there were 3 reported symptomatic non-serious adverse events. Of these, one patient had a minor stroke, one a transitory ischemic attack and one had persistent mass effect symptoms due to a giant aneurysm, none of these resulted in subsequent neurological disability.

**Conclusion:**

This long-term follow-up study demonstrates that the FRED and FRED Jr. are safe and effective for the treatment of cerebral aneurysms in the long term, with high rates of complete occlusion and low rates of delayed adverse events.

## Introduction

The use of flow diverter (FD) stents has become a well-established endovascular technique for the treatment of intracranial aneurysms. The Flow Re-Direction Endoluminal Device (FRED; MicroVention, Aliso Viejo, CA, USA) and its version dedicated for smaller vasculature FRED Jr. (MicroVention) belong to the available FDs with European CE approval. The FRED and FRED Jr. have a dual-layer design with a more porous stent-like outer layer and a low permeable flow diverter part inside the central part of the stent. Several studies showed a good efficacy of the FRED with a good safety profile in the short and mid-term with follow-up raging up to 5 years [1–3]. Analogous results with a follow-up period up to 3 years have been reported for the FRED Jr. [4]; however, to date there are no targeted long term-follow-up data covering more than 5 years for these devices.

In this single-center study we aim to provide long-term safety and efficacy results of aneurysms treated with both FRED and FRED Jr. focusing on patients with follow-up periods of ≥ 5 years. We also examined whether the key variables identified as predictive of aneurysm occlusion in a recent meta-analysis demonstrated similar predictive abilities in our own study [5].

## Material and Methods

### Patient Selection and Data Collection

This study was approved by the local ethics committee. Informed consent was obtained from all subjects before treatment.

Out of our prospectively maintained institutional neurointerventional database we retrospectively identified all consecutive patients who underwent FRED or FRED Jr. implantation for the purpose of cerebral aneurysm treatment between 2013 and 2017. Out of this cohort, only patients with a follow-up imaging greater than 2 years after the FD implantation were analyzed. Both ruptured and unruptured aneurysms were included in this study. Data collection included patient demographics (sex, age and comorbidities); clinical presentation; aneurysm features (type, size, location), vessel diameter and side branches, details of the endovascular treatment, including the pharmacological management, follow-up imaging and clinical outcome.

### Antiplatelet Therapy

The standard antiplatelet therapy included daily dual antiplatelet therapy (DAPT) with 100 mg of aspirin (ASS) and 75 mg of clopidogrel (loading dose, 300 mg), starting no less than 5 days before the procedure. At the day of hospital admission all patients received preprocedure platelet reactivity testing using light transmission aggregometry (LTA) and in case of a drug resistance the medication was changed to either prasugrel or ticagrelor with decisions on a case by case basis. The DAPT with the most appropriate medication was then maintained in the majority of patients for a minimum of 3–6 months after the procedure. After 3–6 months, all patients with a few exceptions were switched to ASS only for additional 6–8 months until reaching 12 months follow-up. Patients with acutely ruptured aneurysms were treated in an emergency setting and received periprocedural and postprocedural i.v. tirofiban (dosage adapted to the patient’s weight according to the instruction for use), followed by the DAPT medication scheme used in the elective patients.

### Evaluation of End Points

#### Efficacy

Imaging analysis was performed by 2 authors with 4 and 8 years of experience in diagnostic neuroradiology, respectively. The follow-up imaging modality of choice at our site is magnetic resonance imaging (MRI) and our protocol foresees sequences to evaluate the brain parenchyma (T1 MPRAGE, T2, FLAIR, SWI, DWI and ADC) and sequences to evaluate the vasculature (precontrast and postcontrast TOF). A DSA during the follow-up is performed only if patients have any clinical conspicuities, the evaluation of the aneurysm is for any reason insufficient with the MRI, the patient has any contraindication for an MRI or if the patient needs a DSA for other reasons (i.e., multiple aneurysms). Radiological follow-up was scheduled after 3, 6, 12 and 24 months and on a 2-year basis thereafter. Aneurysm occlusion rates were assessed using the O’Kelly Marotta scale (OKM) [6] or Raymond-Roy occlusion classification (RROC) [7], for catheter angiography and contrast enhanced MRI scans, respectively. To enhance clarity and comparability for the follow-up assessments of aneurysm occlusion, which were predominantly based on MRI, we referred to the RROC scale. The primary treatment efficacy end point was complete and near-complete occlusion (RROC 1 and 2) at follow-up after 2, 3, 4 and ≥ 5 years. Patients requiring retreatment were specifically assessed.

We additionally performed a dedicated analysis of patients with a follow-up of 5–8 years to focus on long-term safety and efficacy.

#### Safety

All treatment-related complications were recorded during the whole follow-up period. Complications were divided into periprocedural and early complications (≤ 2 years after the treatment) and complications which occurred after 2 years. The primary end point for clinical safety was the absence of treatment-related mortality, symptomatic stroke (major or minor) or transient ischemic attack (TIA) consistent with the vascular territory of the implanted FD, stent patency, and intracranial hemorrhage (ICH). The clinical evaluation was performed by a certified neurologist using the modified Ranking scale (mRS) before treatment, at discharge and during follow-up. A score of 0 and 1 was assumed as a good functional outcome. For mRS scores ≥ 2 we further distinguished if there was a shift between the preprocedural and postprocedural mRS.

### Statistics

SPSS Statistics, Version 25.0 (IBM, Armonk, NY, USA) was used for statistical analysis. Quantitative data are presented as number (relative frequency) or mean ± standard deviation (SD). A linear regression analysis was utilized to evaluate how the rate of complete aneurysm occlusion (dependent variable) is impacted by the age of the patients, the maximum diameter of the aneurysm, and the absence of a side branch originating from the aneurysm sac (independent variables). Values of 0.05 were defined as the threshold for statistical significance and were not adjusted for multiple testing due to the hypothesis-generating fashion of the study. Hence, the *P*-values should be interpreted descriptively.

## Results

### Patient and Aneurysm Characteristics

The main patient and aneurysm characteristics are summarized in Table 1. A total of 75 patients with 91 aneurysms were treated with FRED or FRED Jr. from 2013 to 2017 at our high-volume neurovascular center. After excluding patients with a radiological follow-up less than to 2 years, 68 patients (79% female; mean age 56 ± 12.9 years) harboring 84 aneurysms were included in this study. Of the aneurysms 11 (12%) were ruptured at presentation, out of these 6 were treated with flow diversion in an acute setting, 10 aneurysms (12%) were secondarily treated, due to residual aneurysm filling or regrowth after previous treatment with either coils (*n* = 7), stent-assisted coiling (*n* = 1), flow diverters other than FRED or FRED Jr. without (*n* = 1) or with adjunctive coiling (*n* = 1).

**Table 1:** Main patient, aneurysm and procedural characteristics

*Patient characteristics*				
No. of patients	68			
Patient age	56 ± 12 years (14–80 years)			
Gender	Female: 54 (79%)		Male: 14 (21%)	
Comorbidities	Arterial hypertension: 43 (63%)	DM II: 5 (7%)	Hypercholesterinemia: 30 (44%)	Declared smokers: 28 (41%)
*Aneurysm characteristics*				
No. of aneurysms	84			
Aneurysm location	Extradural ICA: 9 (11%)	Intradural ICA: 67 (80%)	ACA and ACOM: 7 (8%)	Vertebrobasilar: 1 (1%)
Morphology	Saccular: 77 (92%)	Fusiform: 1 (1%)	Dissecting: 1 (1%)	Blister: 5 (6%)
SAH	Non-ruptured: 73 (87%)	Acute: 6 (7%)	Old (> 2 weeks): 5 (6%)	
Aneurysm size (maximum diameter)	7.9 ± 6.7 mm (1.5–31.3 mm)			
Diameter of the parent artery	Proximal: 3.3 ± 0.7 mm (1.6–5 mm)		Distal: 2.9 ± 0.6 (1.5–4.7)	
*Procedural details*				
No. of procedures	70			
No. of implanted FRED/FRED Jr	72			
Technique	FD only: 55 (79%)		FD + coiling: 15 (21%)	

*DM II* diabetes mellitus, type II; *ICA* internal carotid artery; *ACA* anterior cerebral artery; *ACOM* anterior communicating artery; *SAH* subarachnoid hemorrhage; *FD* flow diverter

### Occlusion and Recurrence Rates at Follow-up

**Fig. 1:**
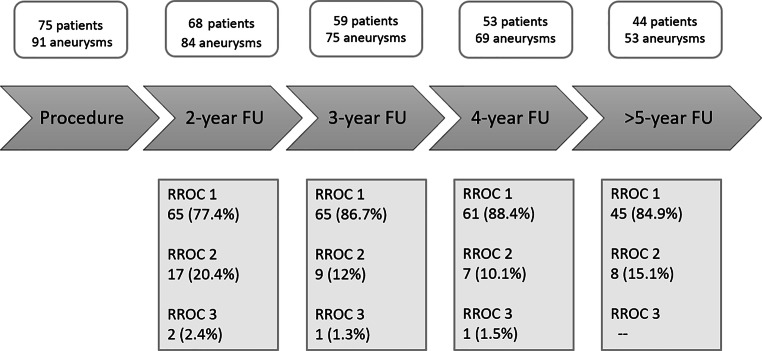
Follow-up time chart. *Above* indicates the number of patients with respective aneurysms for each follow-up milestone; *below* the degree of aneurysm occlusion for each follow-up milestone is indicated by using the Raymond-Roy occlusion classification (RROC). *FU* follow-up

**Fig. 2:**
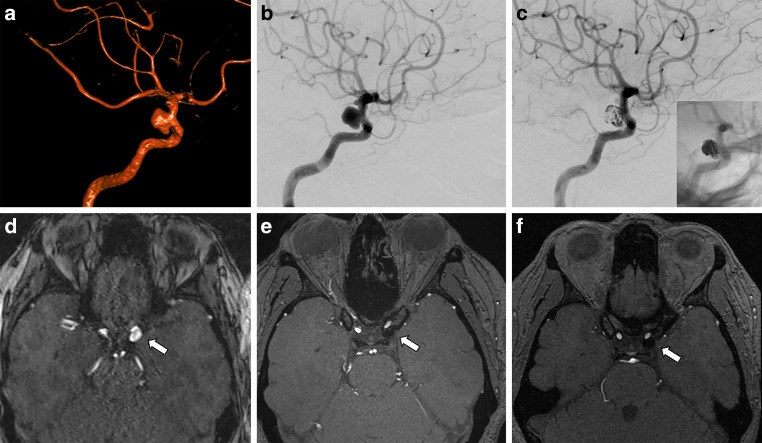
**a** 3D-angiography showing a saccular multilobulated aneurysm of the left internal carotid artery. **b** Subtracted digital subtraction angiography (DSA) before endovascular treatment. **c** Subtracted and unsubtracted DSA after coiling and FRED implantation showing residual aneurysm filling. **d** Magnetic resonance imaging (MRI) time of flight angiography (TOF) angiography before treatment with an arrow pointing to the aneurysms. **e** 1-year follow-up MRI TOF-angiography showing punctate residual perfusion of the aneurysm neck. **f** 6-year follow-up MRI TOF-angiography showing stable complete occlusion of the aneurysm

**Fig. 3:**
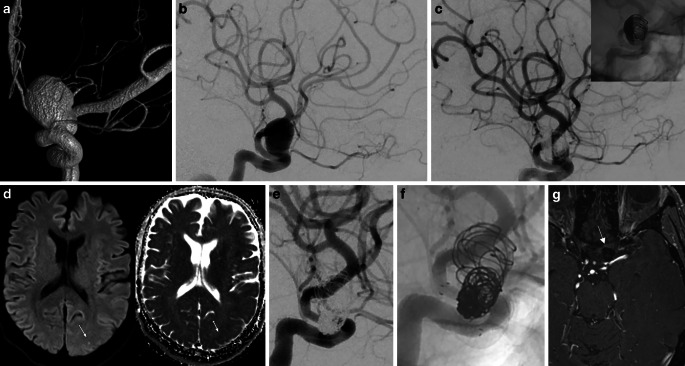
**a** 3D-angiography showing a large (maximum diameter 14 mm) saccular aneurysm in the paraophthalmic left ICA. **b** Subtracted DSA before endovascular treatment. **c** Subtracted and unsubtracted DSA after coiling and FRED implantation showing stasis in the aneurysm. **d** MRI scan after 24.9 months from the index procedure due to several episodes of amaurosis fugax while being under ASS monotherapy: DWI and ADC maps showing a punctate diffusion restriction (arrows) restriction in the left occipital lobe. **e** , **f** Subtracted and unsubtracted DSA of the same day showing the patency of the FD and adjacent vessels, very mild intimal hyperplasia, along with stable complete occlusion of the aneurysm. **g** MRI TOF sequence showing complete aneurysm occlusion at the 5‑year follow-up

Radiological follow-up results are summarized in Fig. 1. Example cases are shown in Fig. 2 and 3.

Follow-up imaging was available in 68 patients (90.7%) with 84/91 aneurysms (92.3%) up to 93.6 months (ca. 8 years) posttreatment (mean 57.3 months; median 57.9 months, both ca. 5 years). The predominant follow-up imaging modality was contrast-enhanced MRI; 6 patients received a DSA at the 3/6 months follow-up and were then examined with MRI thereafter. The 2‑year follow-up showed complete aneurysm occlusion (RROC 1) in 65 aneurysms (77%), residual neck filling (RROC 2) in 17 (20.4%) aneurysms and aneurysmal filling (RROC 3) in 2 (2.4%) aneurysms. Therefore, the primary treatment efficacy end point was reached in 97.8% (RROC 1: 77.4%; RROC2: 20.4%) at 2 years and increased to 100% (RROC 1: 84.9%; RROC 2: 15.1%) when the latest available imaging result is considered.

In a subgroup analysis of occlusion rates during the last follow-up concerning FD type, aneurysms treated with FRED demonstrated complete occlusion (RROC 1) in 87% of cases, residual neck filling (RROC 2) in 10% of cases, and aneurysmal filling (RROC 3) in 3% of cases. Aneurysms treated with FRED Jr. exhibited a RROC 1 occlusion rate in 60% of cases and RROC 2 in 40% of cases, with no instances of residual aneurysm filling.

There were no cases of aneurysm regrowth and no retreatment was necessary for any aneurysm.

The linear regression analysis revealed a statistically significant impact of the patient age (odds ratio, OR, 2.572; 95% CI, 0.002–0.013; *P* = 0.012) and the absence of a side branch originating from the aneurysm sac (OR, 2.036; 95% CI, 0.006–0.488; *P* = 0.045) as well as a notable trend concerning the maximum diameter of the aneurysm (OR, 1.927; 95% CI, 0.000–0.021; *P* = 0.058) on the rates of radiological complete aneurysm occlusion.

### Periprocedural and Early Complications

In the periprocedural and in the period up to the 2‑year threshold we encountered predominantly thromboembolic complications. We observed two cases of acute intraprocedural thrombosis, both of which could be successfully treated by intraprocedural administration of tirofiban followed by a maintenance dose in the following hours after the procedure according to our internal protocol [8]. These patients had no neurological sequelae. One patient developed an early postprocedural occlusion of a side-branch originating from the aneurysm neck which led to a hemorrhagic infarction. This patient recovered overall well with an mRS 1 at the 1‑year follow-up. Another patient suffered from an in-stent thrombosis after 3 months from the index procedure. The vessel could be successfully recanalized by mechanical thrombectomy. Furthermore, we observed one case of asymptomatic parent artery occlusion at the 18-month follow-up and 2 patients suffered from TIAs, at 8 and 19 months after the procedure, respectively.

Besides the thromboembolic complications, we encountered one hemorrhagic complication (groin hematoma), one technical procedural complication (intraprocedural arterial dissection in the ICA, treated with two solitaire stents) and one case of suspected non-ischemic cerebral enhancing (NICE) lesions.

### Delayed Procedure-related Complications and Clinical Outcome

After the 2‑year threshold there were 3 reported symptomatic cases, none of these were serious adverse events with subsequent neurological disability. Two patients had TIAs, one of them with an associated minor stroke, and one had persistent mass effect symptoms due to a giant aneurysm.

Patient no. 1 was treated with FRED and adjunctive coil embolization for a large (maximum diameter 14 mm) saccular aneurysm in the paraophthalmic left internal carotid artery (ICA). After 24.9 months from the index procedure, he reported several episodes of amaurosis fugax while being under ASS monotherapy. A brain MRI showed a punctate diffusion restriction in the left occipital lobe demonstrating a minor stroke. A subsequent diagnostic subtraction angiography (DSA) showed the patency of the FD and adjacent vessels. Also, no intimal hyperplasia was visible. The patients drug regimen was changed to a clopidogrel monotherapy which he did not tolerate due to excessive fatigue. After being switched back to ASS he had another episode of TIA after 3 months. At this time no ischemia could be documented in a brain MRI. A ticagrelor monotherapy was then initiated which was well tolerated by the patient. After 2 further years of follow-up there were no further episodes of TIA. Figure 3 briefly illustrates the case.

Patient no. 2 was treated with a FRED for a saccular ICA aneurysm. After 75.4 months from the index procedure the patient suffered a TIA with symptoms consistent with the vascular territory distal to the aneurysm. A brain MRI showed no ischemic lesions. At this time the patient did not take any antiplatelet or anticoagulant therapy. Therefore, ASS monotherapy was initiated, and the patient did not have any further TIAs up to the latest follow-up.

Patient no. 3 had a giant (maximum diameter 22 mm) aneurysm of the left ICA treated with FRED and coil embolization. The symptoms of double vision and eye motility disorders first worsened postprocedurally, probably due to the extensive intrasaccular thrombosis, and then improved after corticosteroid treatment, however, mild symptoms persisted over the years and acutely worsened 72.1 months after the index procedure. A brain MRI revealed a punctate diffusion restriction in the left parietal lobe which was most likely unrelated to the ophthalmologic symptoms. A following DSA showed a patent stent with no intimal hyperplasia and a fully occluded aneurysm (OKM D). The patient continued ASS monotherapy and was asymptomatic thereafter.

There were no observed cases of delayed aneurysm rupture, subarachnoid or intraparenchymal hemorrhage and no procedure-related deaths in our study. No changes in the mRS scores of the clinical follow-up after 2 years were recorded.

### Patients with Follow-up ≥ 5 Years

A total of 44 patients (80% female; mean age 55 ± 11.7 years) with 53 aneurysms had a radiological and clinical follow-up ≥ 5 years (mean duration 69.2 months; median 68.8 months). After 5 years 45 aneurysms (85%) were completely occluded (RROC 1) and 8 aneurysms (15%) had a residual neck filling (RROC 2). There were no cases of residual aneurysmal filling (RROC 3) or of reperfusion. As mentioned above, 1 case of TIA was recorded after 75.4 months (6.3 years) in patient no. 2. No additional complications were recorded after 5 years.

## Discussion

In this study, 84 aneurysms in 68 patients treated with either FRED or FRED Jr. were analyzed for the long-term outcomes with a median follow-up duration of 57.3 months. The follow-up rate was relatively high, since as many as 68 patients out of 75 (91%) had a radiological follow-up greater than to 2 years. Out of these, 44 patients harbouring 53 aneurysms reached a radiological and clinical follow-up ≥ 5–8 years (65%) (mean duration 69.2 months; median 68.8 months). Our examination of long-term angiographic and clinical outcomes suggests that FRED and FRED Jr. are both effective and safe in the long term with a high occlusion rate and no disabling delayed complications.

Early and mid-term safety and efficacy for the FRED [2, 3, 9, 10] and FRED Jr [4, 11] have been extensively investigated in prior studies. To date this is the first dedicated report on long-term outcomes of flow-diversion with FRED and FRED Jr. with a focus on follow-up periods of ≥ 5 years, investigating the durability of treatment effect and long-term complications after a 5-year threshold.

Flow diversion has in the past years been an increasingly used technique in the endovascular management of intracranial aneurysms due to its good safety and efficacy profile. Lately, the application has even been extended to treat aneurysms with complex morphology or off-label locations [12, 13]. The Pipeline flow diverter (PED; Medtronic Neurovascular, Irvine, CA, USA) was one of the first FD to be systematically evaluated in the Pipeline for Uncoilable or Failed Aneurysms (PUFS) study, dedicated to large and giant wide-necked aneurysms of the intracranial ICA [14]. The recently published 5‑year outcomes of the PUFS trial show a high rate of complete aneurysm occlusion reaching up to 95.2% (60/63 subjects) suggesting that this treatment modality might also be effective in the long term [15]. The so far reported long-term outcomes focusing on the FRED are limited to 2 studies from 2020, both reporting predominantly the 3‑year outcomes [2, 3]. Both studies reported similar results to our finding with a rate of sufficient aneurysm occlusion at last evaluation of 95.9% and 91.9%, respectively, and an increase of complete occlusion rates over time.

Studies analyzing the outcomes of flow diversion after 5 years to further evaluate and confirm the durability of treatment effect are still scarce. So far, only one study analyzed outcomes of flow diversion at a single center including the PED, SILK (Balt Extrusion, Montmorency, France), FRED and p64 in 60 patients harboring 69 unruptured aneurysms [16]. This study had a relatively high availability of long-term follow-up with 50% (30 aneurysms) at 72 months and 42% (25 aneurysms) at 84 months. In a subgroup analysis of the aneurysms treated with FRED (20 patients harboring 24 intracranial aneurysms), complete occlusion (OKM D) was achieved in 23 aneurysms (96%) within 12 months [16]; however, it is not clear whether there were any dynamic changes in the subsequent follow-ups in terms of reperfusion.

A recent comprehensive review of the literature identified three primary factors with the most substantial impact on aneurysm occlusion after treatment with FD: the absence of branch involvement, younger age, and aneurysm diameter [5]. To further investigate this aspect, we conducted a linear regression analysis, affirming a statistically significant influence of two variables within our dataset, namely the age and the absence of branch involvement. Age-related atherosclerotic changes and vessel wall abnormalities affecting the treatment effectiveness is a possible explanation for the influence of age on occlusion rates [5]. The absence of a side branch originating from the aneurysm also was found to be a statistically significant predictor for complete aneurysm occlusion. This phenomenon can be attributed to the requirement for sufficient flow in the branch originating from the aneurysm, which is necessary to supply its respective perfusion territory, thereby causing the continued patency of the aneurysm as a side effect [5]. Additionally, the aneurysm diameter exhibited a notable trend in influencing the rates of occlusion. This observation can be elucidated by considering the typically increased flow rate into larger aneurysms. As a result, the effectiveness of the FD could be diminished, leading to a delay in the formation of intra-aneurysmal thrombosis and, consequently, aneurysm obliteration [5]. In our series the observed RROC 1 occlusion rate for aneurysms treated with FRED Jr. at the last follow-up stood at 60%, a notably lower figure compared to aneurysms treated with FRED, which exhibited an RROC 1 rate of 87%. This variation can be primarily attributed to the shorter mean follow-up period for patients treated with FRED Jr. (37.0 months) in contrast to those treated with FRED (58.9 months), compounded by the recognized tendency for FD treatment to enhance occlusion rates over time.

With respect to complications, FD are linked to a high safety profile in the long term with the majority of complications arising in the perioperative or short postoperative period [8, 17]. As short-term complications have been extensively studied in prior publications, we focus the discussion on those complications that occurred after at least 2 years from the index procedure. The PUFS trial for instance showed no delayed serious device-related events after 3 years nor neurological deaths, hemorrhagic or ischemic cerebrovascular events after 6 months [15]. Consistently, the abovementioned study analyzing different FDs simultaneously showed no complications of any kind after 24 months [16]. In our study we did not observe any neurologically disabling complication in the long-term follow-up starting after 24 months; however, we recorded 1 case of minor stroke at 24.9 and 1 case of TIA 75.4 months after the index procedure. Both of them did not evolve to a major stoke and could be successfully pharmacologically managed. Additionally, these observed episodes could in some cases be unrelated to the prior FD treatment as they occurred in patients with a high cardiovascular risk profile. Similarly, in the PUFS trial 2 events of amaurosis fugax were assessed between the 180-day time point and the 3‑year follow-up [18]. As in our cohort, both did not result in permanent neurological sequelae.

As an additional complication, we reported one case of persistent mass effect after treatment with FRED and adjunctive coiling of a giant ICA aneurysm. In our center, aneurysms larger than 10 mm that are treated with flow-diversion are additionally coiled to reduce the risk of recurrence and rupture. This strategy can in rare cases leave the mass effect unchanged or even aggravate the clinical mass effect syndrome as the coils might interfere with aneurysm retraction. The use of FD with adjunctive loose coiling could be a compromise in these cases [19].

Overall, all studies unanimously suggest that the efficacy and the safety of the flow diverter treatment rises over time with a high rate of adequate and moreover stable occlusion at long-term follow-up complemented by an increasing reduction of treatment related adverse.

The main limitations of this study are the monocentric, retrospective design. The FRED and FRED Jr. were not used exclusively in this time frame and the choice of the used device was at the operator’s own discretion, which might cause a partial selection bias. Additionally, there is an unequal distribution between FRED and FRED Jr. This is in part due to the fact that the FRED Jr. was released later than its larger counterpart. A further limitation is that radiological follow-up evaluations are predominately based on MRA with only a minority of patients having received a long-term DSA. A small number of the patients (*n* = 7) were lost to follow-up in the first 2 years after the respective procedure, which could determine a potential bias in missed early to mid-term adverse events. No specific reason was found for the loss of follow-up in this patient collective.

## Conclusion

This long-term follow-up study suggest that the FRED and FRED Jr. are effective and safe for the treatment of cerebral aneurysms in the long term, with high rates of stable complete occlusion and low rates of delayed adverse events.

## Abbreviations

ACA: Anterior cerebral artery
ACOM: Anterior communicating artery
ADC: Apparent diffusion coefficient
ASS: Aspirin
DAPT: Dual antiplatelet therapy
DM II: Diabetes mellitus, type II
DSA: Diagnostic subtraction angiography
DWI: Diffusion weighted imaging
FD: Flow diverter
FLAIR: Fluid-attenuated inversion recovery
FRED: Flow Re-Direction Endoluminal Device
FRED Jr.: Flow Re-Direction Endoluminal Device Junior
ICA: Internal carotid artery
MRI: Magnetic resonance imaging
mRS: Modified Rankin scale
OKM: O’Kelly Marotta scale
PED: Pipeline flow diverter
PUFS: Pipeline for Uncoilable or Failed Aneurysms study
RROC: Raymond-Roy occlusion classification
SAH: Subarachnoid hemorrhage
SWI: Susceptibility weighted imaging
T1 MPRAGE: T1 Magnetization Prepared – RApid Gradient Echo
TIA: Transient ischemic attack
TOF: Time of flight angiography
